# Pivotal Role of Iron Homeostasis in the Induction of Mitochondrial Apoptosis by 6-Gingerol Through PTEN Regulated PD-L1 Expression in Embryonic Cancer Cells

**DOI:** 10.3389/fonc.2021.781720

**Published:** 2021-11-03

**Authors:** Nipin Sp, Dong Young Kang, Eun Seong Jo, Jin-Moo Lee, Se Won Bae, Kyoung-Jin Jang

**Affiliations:** ^1^ Department of Pathology, School of Medicine, Institute of Biomedical Science and Technology, Konkuk University, Chungju, South Korea; ^2^ Pharmacological Research Division, National Institute of Food and Drug Safety Evaluation, Osong Health Technology Administration Complex, Cheongju-si, South Korea; ^3^ SK Bioscience, Seongnam-si, South Korea; ^4^ Department of Chemistry and Cosmetics, Jeju National University, Jeju, South Korea

**Keywords:** embryonic CSC, 6-gingerol, iron metabolism, PTEN, PI3K/AKT signaling, p53, PD-L1, miR-20b/miR-21/miR-130b

## Abstract

Embryonic cancer stem cells (CSCs) can differentiate into any cancer type. Targeting CSCs with natural compounds is a promising approach as it suppresses cancer recurrence with fewer adverse effects. 6-Gingerol is an active component of ginger, which exhibits well-known anti-cancer activities. This study determined the mechanistic aspects of cell death induction by 6-gingerol. To analyze cellular processes, we used Western blot and real-time qPCR for molecular signaling studies and conducted flow cytometry. Our results suggested an inhibition of CSC marker expression and Wnt/β-catenin signaling by 6-gingerol in NCCIT and NTERA-2 cells. 6-Gingerol induced reactive oxygen species generation, the DNA damage response, cell cycle arrest, and the intrinsic pathway of apoptosis in embryonic CSCs. Furthermore, 6-gingerol inhibited iron metabolism and induced PTEN, which both played vital roles in the induction of cell death. The activation of PTEN resulted in the inhibition of PD-L1 expression through PI3K/AKT/p53 signaling. The induction of PTEN also mediated the downregulation of microRNAs miR-20b, miR-21, and miR-130b to result in PD-L1 suppression by 6-gingerol. Hence, 6-gingerol may be a promising candidate to target CSCs by regulating PTEN-mediated PD-L1 expression.

## Introduction

Stem cells differentiate into any kind of tissue, and embryonic stem (ES) cells can differentiate into embryonic germ layer derivatives that could generate any kind of tissue present in the human body. Embryonic cancer stem cells (CSCs), however, exhibit these properties in addition to non-restricted proliferation, which makes them more hazardous than other cancer cell types (1, 2). Embryonic CSCs may be able to differentiate into various cancers, such as those of the colon, breast, and lung (3). The sex-determining region Y (SRY)-box 2 (SOX2), octamer-binding transcription factor 4 (OCT4), and homeobox protein NANOG are CSC markers overexpressed in CSC that help initiate tumorigenesis and maintain their pluripotent nature (4, 5). Many additional signaling pathways exist that can promote the self-renewal ability and pluripotency of CSC, among them is the Wnt/β-catenin pathway, which plays a vital role in tumor progression (6, 7). In canonical Wnt signaling, secreted glycoprotein Wnt family proteins and β-catenin, a transcriptional activator for the Wnt family, promote homeostasis and embryonic development (8). The activation of β-catenin is regulated by casein kinase 1α and glycogen synthase kinase 3β (GSK-3β) for proteasomal degradation or ubiquitination, respectively. Activation allows β-catenin to bind a transcription factor (TCF)-binding element of the TCF/lymphoid enhancer-binding factor in the nucleus to initiate transcription (9). Hence, Wnt/β-catenin signaling plays a key role in developing CSCs, which makes Wnt/β-catenin signaling and CSC markers potential targets for effective CSC treatments and possibly other cancer cells (10, 11).

The tumor suppressor phosphatase and tensin homologue (PTEN) is a tumor suppressor with a crucial rule in tumorigenesis of negatively regulating phosphoinositide 3-kinase (PI3K) and protein kinase B (AKT) signaling, which is a key pathway for cancer cell proliferation and survival (12, 13). Loss of PTEN function is considered a major reason for tumorigenesis and has been associated with most cancer types; PTEN mutation causes a disease known as Cowden syndrome (14). During the tumorigenesis stage, a loss of PTEN inhibits p53 signaling through the activation of PI3K/AKT, which then promotes the upregulation of CSC markers such as SOX2 and OCT4, and it activates programmed death-ligand 1 (PD-L1) (15, 16). PD-L1 is overexpressed in many cancer types, and it facilitates immune escape by binding its ligand, PD-1, which is present on the surface of lymphocytes, myeloid cells, T-cells, and B-cells (17–19). Hence, anti-cancer treatments that target PTEN could also lead to the suppression of PD-L1 expression and prevent immunosuppression.

Iron plays a central role in cellular metabolism and is a necessary cofactor in enzymes that mediate cell growth and cell death. Iron-containing proteins regulate mitochondrial functions, DNA synthesis, damage response, and oxygen transport (20). Biomolecule oxidation can generate reactive oxygen species (ROS) that make iron toxic (21). Hence, iron homeostasis must balance the presence of iron with the help of iron channels to prevent excessive ROS. Furthermore, in cancer, iron levels may regulate epigenetic alterations and maintain genomic stability as well as mediate tumor metastasis and the tumor microenvironment (TME) (22). The possibility that iron homeostasis possesses the dual role of cancer cell death and tumor proliferation depends on its specific role in cellular functions (23). Generally, transferrin-iron complexes (Fe3+) enter the cytoplasm *via* the transferrin receptor. Then, the iron is converted into Fe2+ with the help of several enzymes such as six-transmembrane epithelial antigen of prostate 3 (STEAP3) and divalent metal transporter 1 (DMT1), prior to taking part in cellular metabolism and heme biosynthesis (24). Next, this shows the significance of iron metabolism in the body, as their variation may result in inflammation and tumorigenesis.

Patients suffering from cancer also struggle with the adverse effects of chemotherapeutic drugs (25, 26). Cancer treatments that use natural compounds are good alternatives, due to the possibility of a multi-targeted treatment with fewer side effects compared to chemotherapeutics (27, 28). Ginger is a very popular spice commonly used in Asian countries, and 6-gingerol is a bioactive phenolic compound and primary pharmacological component of ginger (29). It exhibits antioxidant, anti-platelet, anti-inflammatory, anti-proliferative, and anti-cancer activities (30–33). 6-Gingerol has been reported to induce anti-tumor activity against breast (28), colorectal (34), gastric (35), and pancreatic cancers (36). However, the mechanism of cell death by 6-gingerol in CSCs is unknown.

This study demonstrates the ability of 6-gingerol to induce apoptosis in NCCIT and NTERA-2 embryonic CSCs and a role for iron metabolism in PTEN-mediated PD-L1 under these conditions. Also, we analyzed the molecular mechanism behind the induction of apoptosis by 6-gingerol in CSCs.

## Materials and Methods

### Antibodies and Cell Culture Reagents

Roswell Park Memorial Institute-1640 (RPMI-1640) medium, penicillin-streptomycin solution, and trypsin-EDTA (0.05%) were purchased from Gibco (Thermo Fisher Scientific, Inc., Waltham, MA, USA). Dulbecco’s modified Eagle’s Media (DMEM; LM001-51) was purchased from Welgene Biotech (Taipei City, Taipei, Taiwan). 6-Gingerol (cat no. 23513-14-6) was purchased from TCI (Tokyo Chemical Industry Co., Tokyo, Japan). Fetal bovine serum (FBS; 12003C) and primary antibodies specific for SOX2 (MAB4423), OCT4 (MABD76), NANOG (MABD24), SF1670 (SML0684), and iron (II) sulfate heptahydrate (F8633) were purchased from Sigma-Aldrich (Merck KGaA, St. Louis, MO, USA). Antibodies specific for β-actin (sc-47778), Wnt5A (sc-365370), BCL-2 (sc-7382), p21 (sc-756), cyclin E (sc-481), and CDK4 (sc-260) and secondary antibodies [anti-mouse (sc-516102) and anti-rabbit (sc-2357)] were obtained from Santa Cruz Biotechnology, Inc. (Dallas, TX, USA). Next, the Wnt8A (H00007478-B01P) antibody was obtained from Abnova (Taipei City, Taiwan). β-Catenin (#9582), GSK-3β (#9315), BAX (#2772), BCL-xL (#2764), cytochrome C (#11940), p27 Kip1 (#3686), p53 (#9282), pATM (#5883), pATR (#2853), pCHK1 (#2348), pCHK2 (#2197), pBRCA1 (#9009), TCF3/TCF7L1 (#2883), Casp9 (#9502), C-Casp9 (#9505), PTEN (#9188), pAKT (#4060), AKT (#4691), pPI3K (#4228), PI3K (#4257), COX IV (#4850), and GAPDH (#2118) antibodies were purchased from Cell Signaling Technology, Inc. (Beverly, MA, USA). TFR1 (ab84036), STEAP3 (ab151566), DMT1 (ab55735), and cyclin D1 (ab6152) antibodies were purchased from Abcam (Cambridge, MA, USA). FPN1 (NBP1-21502) and iNOS (NB300-650) antibodies were obtained from Novus Biologicals (Littleton, CO, USA). Finally, the antibody specific for PD-L1 (R30949) was obtained from NSJ Bioreagents (San Diego, CA, USA).

### Cell Culture and Treatment

NCCIT (CRL-2073) and NTERA-2 (CRL-1973) cell lines were purchased from the American Type Culture Collection (ATCC; Manassas, VA, USA). Next, NCCIT cells were cultured and maintained in RPMI-1640 media, and NTERA-2 cells were cultured and maintained in DMEM media plus 10% FBS and 1% penicillin at 37°C in 5% CO2. The medium was changed three times a week after cells reached up to 80% confluence and treated with 6-gingerol. Lastly, the treated cells were incubated at 37°C for 48 h.

### Cell Viability Assay

Cell viability was measured using a 3-(4,5-dimethylthiazol-2-yl)-2,5-diphenyltetrazolium bromide (MTT) assay. Then, NCCIT or NTERA-2 cells were maintained in culture media in 96-well culture plates at 3 × 10^3^ per well (density) for 24 h. Next, cells were incubated in fresh medium containing dimethyl sulfoxide (DMSO) as the vehicle control and treated with 6-gingerol (50–400 μM) for 48 h. Subsequently, MTT (5 mg/mL) was added and incubated for 4 h at 37°C. The resulting formazan product was dissolved in DMSO, and an Ultra Multifunctional Microplate Reader (Tecan, Durham, NC, USA) was used to measure the absorbance at a wavelength of 590 nm. All measurements and experiments were conducted in triplicate.

### Western Blotting

Protein samples were isolated from untreated (control) or 6-gingerol-treated NCCIT or NTERA-2 cells using radioimmunoprecipitation (RIPA) lysis buffer (20–188; EMD Millipore), which contained protease and phosphatase inhibitors. First, the concentration of proteins was measured using Bradford’s method (Thermo Fisher Scientific). Next, 100 μg of protein from each sample were resolved with sodium dodecyl sulfate-polyacrylamide gel (10%–15%) electrophoresis. Then, the separated proteins were transferred onto nitrocellulose membranes, followed by blocking with 5% skim milk (BD Biosciences, San Jose, CA, USA) in TBS-T buffer [20-μM Tris-HCl (Sigma-Aldrich; Merck KGa A), pH 7.6, 137-μM NaCl (Formedium, Norfolk, UK; NAC03), and 0.1× Tween20 (Scientific Sales, Inc. OakRidge, TN, USA)] for 1 h. Next, the membranes were incubated overnight at 4°C in a shaker with specific primary antibodies diluted in 5% bovine serum albumin (EMDMillipore). Then, the membranes were washed with TBS-T and incubated with HRP-conjugated secondary antibodies for 1 h at room temperature. Finally, the detection was performed using a Femto Clean Enhanced Chemiluminescence Solution Kit (77449; GenDEPOT, Katy, TX, USA) in a LAS-4000 imaging device (Fujifilm, Tokyo, Japan). Quantifications were conducted using ImageJ software (v.1.8.0_172; National Institutes of Health).

### Quantitative Real-Time Polymerase Chain Reaction (qPCR)

Total RNA was isolated using the RNeasy Mini Kit (Qiagen GmbH, Hilden, Germany) and then quantified using a spectrophotometer at 260 nm. A thermal cycler (C1000 Thermal Cycler; Bio-Rad, Hercules, CA, USA) was used to make cDNA from the total RNA using a first-strand cDNA synthesis kit (Bioneer, Daejeon, Korea) and oligo (dT) primers. PD-L1, p53, and GAPDH cDNA (2-5 µg) were amplified using an RT-PCR Premix Kit (Bioneer) with primers synthesized by Bioneer. The Light Cycler 480II (Roche) was used for qPCR as follows: 2 μL of diluted cDNA was mixed with 10 μL of TB Green Advantage Premix (Takara Bio, Japan) and 1 μL each of the forward and reverse primers. The cycling conditions were as follows: 95°C for 5 min for the initial denaturation, which was followed by 40 cycles of 95°C for 40 s, 58°C for 40 s, 72°C for 40 s, and a final extension of 5 min at 72°C. All reactions were conducted three times and normalized to GAPDH; quantifications were conducted using the obtained Cp values.

### FACS Analysis for Mitochondrial Membrane Potential and ROS

The cultured cells were washed with pre-warmed culturing medium supplemented with 10% FBS (staining buffer), and 1 × 10^6^ cells were re-suspended in 1 mL of staining buffer containing MitoTracker Deep Red (40 nM; Invitrogen, Carlsbad, CA, USA: M22426) to measure mitochondrial membrane potential, MitoSOX (5 μM; M36008; Invitrogen) to measure mROS, or CM-H2DCFDA (5 μM; Invitrogen, C6827) to measure cellular ROS. Then, the cells were incubated in a CO2 incubator at 37°C for 30 min, and the stained cells were washed with 1 mL of pre-warmed staining buffer prior to fluorescence-activated cell sorting (FACS). The analysis was performed using FlowJo software.

### Cell Cycle Analysis

The DNA content of 6-gingerol-treated and non-treated cells was determined using a BD Cycletest Plus DNA Reagent Kit (BD Biosciences, San Jose, CA, USA) according to the manufacturer’s protocol. Approximately 5 × 10^5^ cells, incubated with or without 6-gingerol for 48 h, were washed with PBS and permeabilized with trypsin. RNA interactions with propidium iodide (PI) were neutralized by treating the cells with RNase buffer and trypsin inhibitor. Then, the samples were stained with PI and incubated for 30 min in the dark at room temperature prior to analysis with a FACSCalibur flow cytometer (BD Biosciences, San Jose, CA, USA).

### Comet Assay

The comet assay kit (Abcam, Cambridge, MA, USA) was used to measure cellular DNA damage. This assay is a single-cell gel electrophoresis method for the simple evaluation of cellular DNA damage. First, a base layer of comet agarose was created on a slide, followed by a layer of cells, agarose, and lysis. Next, electrophoresis was performed under neutral conditions, and the cells were stained with DNA dye. Finally, cell morphology was observed by fluorescence microscopy (Olympus IX71/DP72).

### Apoptosis Analysis

Fluorescein-conjugated annexin V (annexin V-FITC) was used to measure apoptosis in NCCIT and NTERA-2 cells. First, the 6-gingerol-treated or untreated cells were washed with PBS and re-suspended in a binding buffer at a concentration of 1 × 10^6^ cells. Then, the cells were stained with annexin V-FITC and PI for 10 min in a dark room at room temperature. Finally, the percentage of apoptotic cells was measured by flow cytometry *via* FACSCalibur, and the analysis was performed using FlowJo software.

### Isolation of Mitochondria/Cytosol Fractions

Mitochondria/cytosol fractions from 6-gingerol-treated and non-treated NCCIT and NTERA-2 cells were extracted using a mitochondria/cytosol fractionation kit (Abcam). A 1X cytosol extraction buffer containing DTT and protease inhibitors was added to the cells (5 x 10^7^), which were then incubated on ice for 10 min. After incubation, samples were centrifuged at 700 x g for 10 min at 4°C to collect the supernatant, which was centrifuged again at 10,000 x g for 30 min at 4°C. The supernatant was removed and saved as the cytosol fraction, while the pellet was re-suspended in PBS to obtain the mitochondrial fraction. Next, western blotting of cytochrome c was carried out as described above.

### ATP Determination Assay

The ATP Determination Kit (Molecular Probes, Eugene, OR, USA) was used to measure ATP. Briefly, NCCIT or NTERA-2 cells were treated with 6-gingerol, and an equal number of cells was collected for the ATP determination assay. The standard reaction solution was made using reaction buffer, DTT, D-luciferin, and firefly luciferase as provided in the kit; then, cells were added along with the standard reaction solution. Luminescence readings were taken immediately using a plate-reading luminometer, and calculations were performed according to the assay protocol.

### Iron Estimation Assay

Iron estimation was performed using an iron assay kit (MAK025) purchased from Sigma-Aldrich (Merck KGaA, St. Louis, MO). Briefly, NCCIT or NTERA-2 cells (2 × 10^6^) treated with or without 6-gingerol were homogenized in an iron assay buffer and collected by centrifugation at 16,000 × g for 10 min at 4°C. Then, the cells were mixed with an iron assay buffer, added to a 96-well plate along with an iron reducer, and incubated in a horizontal shaker for 30 min at 25°C. Then, 100 µL of the iron probe was added to each well and incubated for 1 h at 25°C. Absorbance was measured at 593 nm, and controls were set to 100% for comparison.

### FACS Analysis for Ferrous Iron (Fe^2+^)

After cultured cells (NCCIT or NTERA-2) were washed with culturing medium, 2 mL of staining solution containing FerroFarRed (5 µM; GC903-01; GORYO Chemical) was added prior to incubation in a CO2 incubator at 37°C for 30–40 min. After staining, cells were washed with 1 mL of pre-warmed serum-free culture medium and used for FACS analysis.

### Statistical Analyses

All experiments were performed in triplicate. The results were expressed as the mean ± standard error of the mean. Statistical analyses were conducted *via* the one-way analysis of variance (ANOVA) or Student’s t-test. Additionally, the one-way ANOVA was performed using Tukey’s *post hoc* test. The analyses were performed using SAS 9.3 software (SAS Institute, Inc., Cary, NC, USA). A p-value < 0.05 (*) was considered statistically significant.

## Results

### 6-Gingerol In hibits CSC Markers and Wnt/β-catenin Signaling in Embryonic CSCs

**Figure 1:**
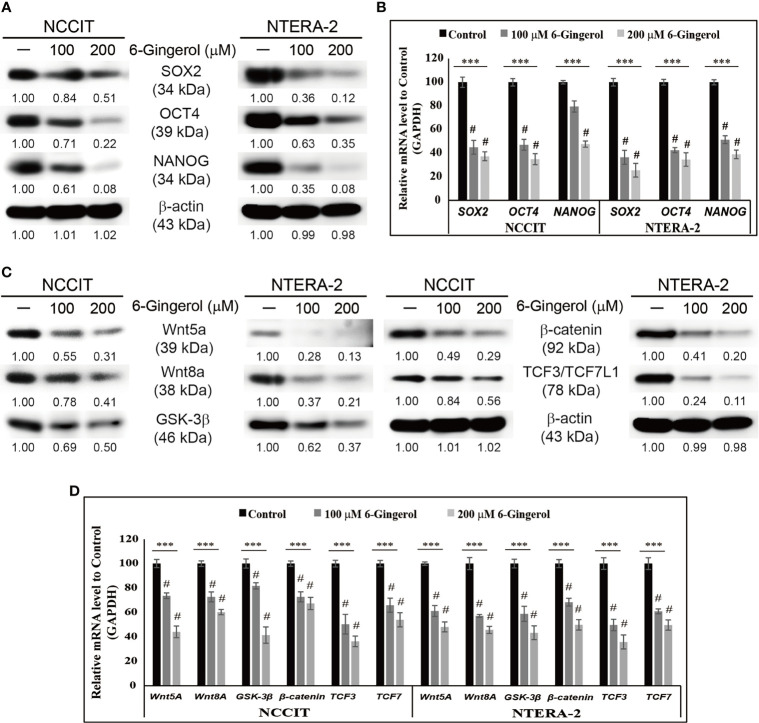
Inhibition of CSC markers and Wnt/β-catenin signaling by 6-gingerol. **(A)** Western blot analysis of SOX2, OCT4, and NANOG in NCCIT and NTERA-2 cells after treatment with 100 or 200 μM 6-gingerol for 48 h. Expression levels of proteins were estimated by densitometry and normalized to β-actin. Data were obtained in triplicate. **(B)** Real-time qPCR showing illus-trative expression of *SOX2*, *OCT4*, and *NANOG* genes in embryonic CSCs. The obtained Cp values were normalized to *GAPDH* mRNA. Controls were set at 100. ***p < 0.001 (ANOVA). ^#^ *p* < 0.001 *vs*. control. **(C)** Western blot of Wnt5a, Wnt8A, GSK-3β, β-catenin, and TCF3/TCF7L1 in NCCIT and NTERA-2 cells after treatment with 100 or 200 μM 6-gingerol for 48 h. Expression levels were normalized to β-actin. Experiments were conducted three times for confirmation. **(D)** Real-time qPCR showing illustrative expression of *Wnt5a*, *Wnt8A*, *GSK-3β*, *β-catenin*, *TCF3*, and *TCF7* genes in NCCIT and NTERA-2 cells. Cp values were normalized to *GAPDH* mRNA. Controls were set at 100. ***p < 0.001 (ANOVA). ^#^ *p* < 0.001 *vs*. control.

To begin our study, first, we analyzed the proliferation inhibition of 6-gingerol on embryonic CSC viability by the MTT assay. Increasing concentrations of 6-gingerol in NCCIT and NTERA-2 (**Supplementary Figure 1A**) resulted in a concentration-dependent inhibition of cell viability. From these results, we calculated 200 μM 6-gingerol to be the IC_50_ dosage and selected 100 or 200 μM of 6-gingerol for concentration-dependent studies. We also evaluated the cell morphology of embryonic CSCs after treatment with 6-gingerol using DAPI staining. We observed a decrease in cell number upon 6-gingerol treatment (**Supplementary Figure 1B**). Next, we determined whether 6-gingerol could suppress CSC markers in embryonic CSCs. Evaluation of the CSC markers SOX2, OCT4, and NANOG in embryonic CSCs with 6-gingerol treatment showed a downregulation in the expression patterns of these CSC markers (**Figure 1A**). Then, we confirmed their downregulation in NCCIT and NTERA-2 cells at the mRNA level using real-time qPCR analysis (**Figure 1B**). These results also suggested the ability of 6-gingerol to inhibit CSC proliferation.

Next, we analyzed the expression patterns of Wnt/β-catenin signaling in embryonic CSCs following 6-gingerol treatment. The results showed a downregulation in the expression of Wnt5A, Wnt8A, GSK-3β, β-catenin, and TCF proteins by 6-gingerol treatment in NCCIT and NTERA-2 cells (**Figure 1C**). Then, we confirmed the inhibition of Wnt/β-catenin signaling at the mRNA level (**Figure 1D**). These results suggested the capability of 6-gingerol against embryonic CSCs. We also analyzed the ability of 6-gingerol to inhibit tumor invasion in embryonic CSC using the Matrigel invasion assay. Results indicated a successful inhibition of tumor invasion in NCCIT and NTERA-2 cells by 6-gingerol treatment (**Supplementary Figure 1C**). These results suggested the overall cytotoxic activity of 6-gingerol against embryonic cancer stem cells.

### 6-Gingerol Induces ROS and DNA Damage Response in Embryonic CSCs

**Figure 2:**
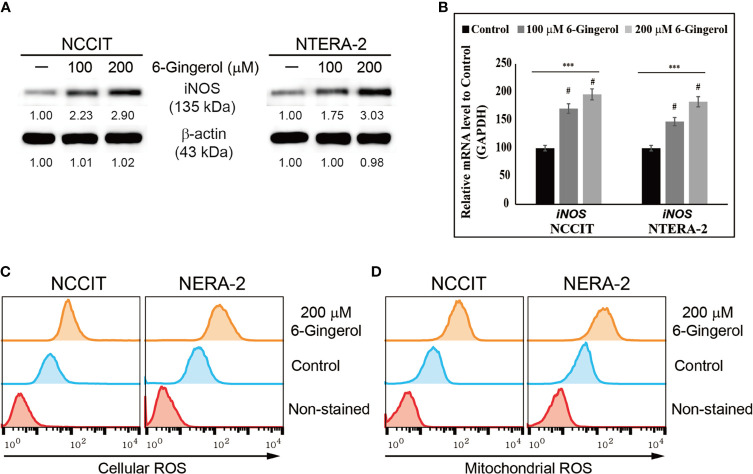
6-Gingerol induced ROS formation in embryonic CSCs. **(A)** Western blot of iNOS protein expression in NCCIT and NTERA-2 cells incubated with 100 or 200 μM 6-gingerol for 48 h. Expression levels were estimated by densitometry and normalized to β-actin. Data were obtained in triplicate. **(B)** Real-time qPCR analysis showing illustrative expression of iNOS in NCCIT and NTERA-2 cells. Next, the obtained Cp values were normalized to *GAPDH* mRNA. Controls were set at 100. ***p < 0.001 (ANOVA). ^#^ *p* < 0.001 *vs*. control. **(C)** Flow cytometry of cellular ROS in NCCIT and NTERA-2 cells incubated with 200 μM of 6-gingerol for 48 h. The graphical representation shows cells with ROS induction. **(D)** Flow cytometry of mitochondrial ROS following 200 μM 6-gingerol treatment of NCCIT and NTERA-2 cells for 48 h. The graphical representation shows cells with mitochondrial ROS.

We hypothesized that the anti-tumor activity of 6-gingerol might begin by generating ROS. To analyze this, we measured iNOS expression following 6-gingerol treatment. Our results showed that increasing concentrations of 6-gingerol upregulated iNOS expression in NCCIT and NTERA-2 cells (**Figure 2A**). We confirmed this by measuring the expression of *iNOS* mRNA level in embryonic CSCs after treatment with 6-gingerol with similar results (**Figure 2B**). The induction of iNOS suggested that 6-gingerol may cause ROS generation in embryonic CSCs. As we expected, we found that 6-gingerol treatment successfully induced cellular (**Figure 2C**) and mitochondrial (**Figure 2D**) ROS, which suggested ROS as the reason for the anti-tumor activity of 6-gingerol.

Then, we verified the ability of 6-gingerol to induce the DNA damage response (DDR) in NCCIT and NTERA-2 cells. To analyze this, we used a comet assay to determine DNA double-strand breaks, which showed that 6-gingerol induced DNA double-strand breaks in NCCIT and NTERA-2 cells (**Supplementary Figure 2A**). Moreover, we observed a significant increase in the comet length and number of comet-positive cells in 6-gingerol-treated cells compared with non-treated control cells (**Supplementary Figure 2B**). These results hinted at the DDR induction of 6-gingerol in embryonic CSCs. To confirm this, we evaluated the expression levels of proteins involved in the DDR and found an increase in the expression of phosphorylated histone, ATM, ATR, CHK1, CHK2, and BRCA1 following 6-gingerol treatment in NCCIT and NTERA-2 cells (**Supplementary Figure 2C**). These results suggested that either ATM or ATR may act as a key regulator of DDR induction by 6-gingerol.

### 6-Gingerol Induces Cell Cycle Arrest and Intrinsic Apoptosis in Embryonic CSCs

Based on previous results, we showed that 6-gingerol induces ROS and the DDR in embryonic CSCs. Therefore, we analyzed the impact of 6-gingerol on the cell cycle and apoptosis induction. Flow cytometry of embryonic CSCs treated with 6-gingerol showed an arrest in the G0/G1 phase of the cell cycle (**Supplementary Figure 3A**). These results indicated that DDR induction leads to prolonged cell cycle arrest, and to confirm this, we analyzed cell cycle checkpoint protein levels by Western blot. These results showed an elevation in the expression of tumor suppressor proteins p21 and p27 as well as a decrease in the expression of cyclin D1, cyclin E, and CDK4 proteins (**Supplementary Figure 3B**). Then, we confirmed these results by measuring the transcriptional expression of *CCND1*, *CCNE1*, *CDK4*, *CDKN1A*, and *CDKN1B* genes (**Supplementary Figure 3C**), which confirmed the induction of a cell cycle arrest. These results indicated that 6-gingerol causes cell cycle arrest in embryonic CSCs and may also induce apoptosis.

**Figure 3:**
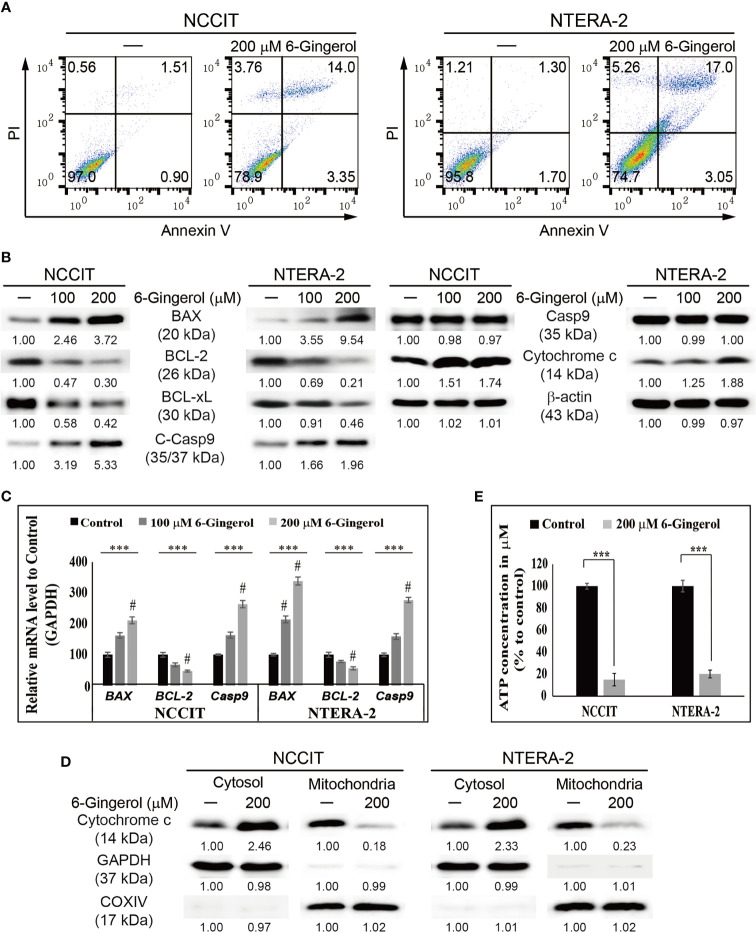
6-Gingerol induced intrinsic apoptosis pathway. **(A)** Fluorescein-conjugated annex-in V (annexin V-FITC) *vs*. propidium iodide (PI) staining analysis in NCCIT and NTERA-2 cells following incubation with 200 μM 6-gingerol for 48 h. **(B)** Western blot of BAX, BCL-2, BCL-xL, C-Casp9, Casp9, and cytochrome c in NCCIT and NTERA-2 cells with 100 or 200 μM 6-gingerol for 48 h. Expression levels were estimated by densitometry and normalized to β-actin. Data were obtained in triplicate. **(C)** Real-time qPCR analysis showing illustrative expression of *BAX*, *BCL-2*, and *caspase 9* genes in NCCIT and NTERA-2 cells incubated with 100 or 200 μM 6-gingerol for 48 h. Next, the obtained Cp values were normalized to *GAPDH* mRNA. Controls were set at 100. ***p < 0.001 (ANOVA test). ^#^ *p* < 0.001 *vs*. control. **(D)** Western blot of cyto-chrome c in cytosolic and mitochondrial fractions isolated from NCCIT and NTERA-2 cells after 48 h treatment with 200 μM of 6-gingerol. *GAPDH* and COXIV were the controls for cyto-solic and mitochondrial fractions, respectively. **(E)** A plot of the ATP concentration following treatment with 200 μM of 6-gingerol in NCCIT and NTERA-2 cells. Controls were set at 100. ***p < 0.001 (Student’s t-test).

Next, we evaluated apoptosis induction by 6-gingerol in embryonic CSCs using flow cytometry. The results showed that 6-gingerol induced apoptosis in NCCIT and NTERA-2 cells (**Figure 3A**). Then, we investigated the apoptosis pathway by measuring protein levels of the key apoptosis regulators BCL2 associated X (BAX), B-cell lymphoma 2 (BCL-2), B-cell lymphoma-extra-large (BCL-xL), cleaved caspase 9, and cytochrome c (**Figure 3B**). These results showed a downregulation in the expression levels of BCL-2 and BCL-xL while BAX, cleaved caspase 9, and cytochrome c were upregulated upon 6-gingerol treatment. These data suggested a possible induction of the intrinsic apoptosis pathway. As intrinsic apoptosis depends heavily on the BAX/BCL-2 ratio, we confirmed the expression of *BAX*, *BCL-2*, and *CASPASE 9* mRNA following 6-gingerol treatment in embryonic CSCs (**Figure 3C**). These results suggested a possible release of cytochrome c from the mitochondria into the cytosol, which was confirmed by comparing cytochrome c levels in the cytosol and mitochondria (**Figure 3D**). These results showed a decrease in the amount of cytochrome c in mitochondria and a corresponding increase in the cytosol. Also, we analyzed the ATP concentration of embryonic CSCs after treatment with 6-gingerol (**Figure 3E**); the results indicated a decrease in ATP production, which corresponds with a 6-gingerol induction of the intrinsic apoptosis pathway.

### 6-Gingerol Regulates PTEN-Mediated PD-L1 Expression in Embryonic CSCs

**Figure 4:**
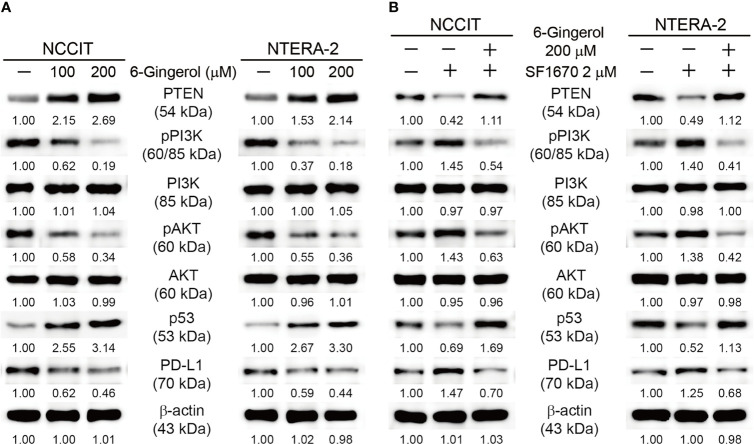
6-Gingerol regulated PTEN/PD-L1 expression. **(A)** Western blot of PTEN, phos-pho-PI3K, PI3K, phospho-AKT, AKT, p53, and PD-L1 in NCCIT and NTERA-2 cells incubated with 100 or 200 μM 6-gingerol for 48 h. Expression levels were estimated by densitometry and normalized to β-actin. Data were obtained in triplicate. **(B)** Western blot of PTEN, phos-pho-PI3K, PI3K, phospho-AKT, AKT, p53, and PD-L1 in NCCIT and NTERA-2 cells incubated with 2 μM SF1670 or 200 μM 6-gingerol for 48 h. Expression levels were estimated by densi-tometry and normalized to β-actin. Data were obtained in triplicate.

Taken together, these data indicate the induction of intrinsic apoptosis by 6-gingerol in embryonic CSCs. To identify the molecular signaling responsible for this mechanism, we analyzed the expression of tumor suppressor protein PTEN and its downstream targets PI3K, AKT, and p53. We found elevated expression of PTEN and p53 while phosphorylated PI3K and AKT were downregulated by 6-gingerol treatment of NCCIT and NTERA-2 cells (**Figure 4A**). Also, we analyzed the expression of PD-L1, as PI3K/AKT/p53 signaling may regulate its expression. Our results suggested the same, as 6-gingerol suppressed the expression levels of PD-L1 in embryonic CSCs. This result hinted at the role of PTEN/PI3K/AKT/p53 signaling in PD-L1 inhibition by 6-gingerol. To confirm this signaling and its effect on PD-L1 expression, we repeated our experiment with a specific inhibitor of PTEN (SF1670). We found increased phospho-PI3K/AKT and PD-L1 expression upon SF1670 treatment, and 6-gingerol successfully inhibited the expression of these proteins (**Figure 4B**). The expression of p53 was suppressed with PTEN inhibitor treatment and rescued by the addition of 6-gingerol in embryonic CSCs, which suggested the ability of 6-gingerol to induce PTEN and p53 expression and thereby inhibit PD-L1 expression. It also suggested a role for PTEN in the regulation of PD-L1 during 6-gingerol treatment.

### 6-Gingerol Induces Iron Release and Inhibits Iron Metabolism in Embryonic CSCs

**Figure 5:**
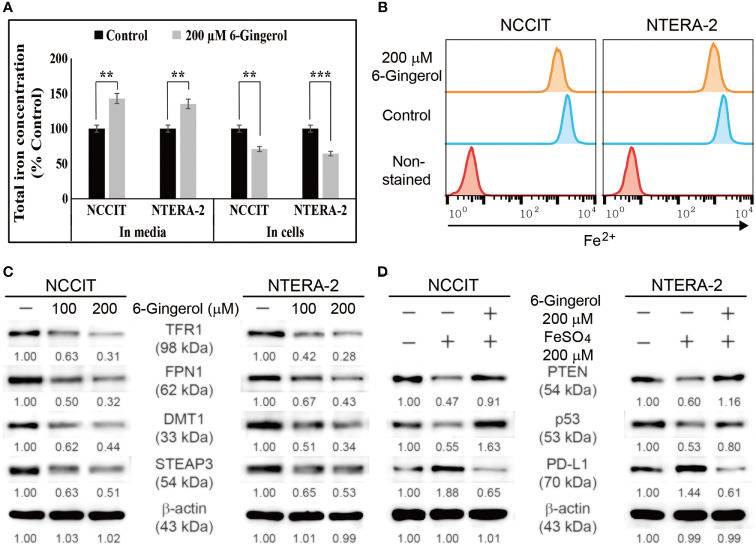
6-Gingerol inhibited iron metabolism. **(A)** Iron assay of the total iron concentration in NCCIT and NTERA-2 cells treated with 200 μM 6-gingerol for 48 h. Data were obtained in triplicate. Controls were set at 100. **p < 0.01 and ***p < 0.001 (control *vs.* 6-gingerol; Student’s t-test). **(B)** Flow cytometry of Fe^2+^ in NCCIT and NTERA-2 cells after treatment with 200 μM of 6-gingerol for 48 h. The graphical representation shows cells with intracellular Fe2+ level. **(C)** Western blot of TFR1, DMT1, STEAP3, and FPN1 in NCCIT and NTERA-2 cells after in-cubation with 100 or 200 μM of 6-gingerol for 48 h. Expression levels were estimated by densi-tometry and normalized to β-actin. Data were obtained in triplicate. **(D)** Western blot of PTEN, p53, and PD-L1 in NCCIT and NTERA-2 cells incubated with 200 μM FeSO_4_ or 200 μM 6-gingerol for 48 h. Expression levels were estimated by densitometry and normalized to β-actin. Data were obtained in triplicate.

We found that 6-gingerol can induce intrinsic apoptosis in embryonic CSCs. Next, we analyzed the mechanism of these activities and hypothesized that iron homeostasis might play a role. First, we estimated the total iron concentration in NCCIT and NTERA-2 cells and media with or without 6-gingerol treatment using an iron assay kit (**Figure 5A**). The results showed an increase in the total iron concentration in the medium following 6-gingerol treatment. However, the concentration of iron in 6-gingerol-treated cells was significantly reduced. This suggested an enhanced iron release into the medium. We confirmed this by using flow cytometry to estimate the ferrous ion (Fe2+) concentration in NCCIT and NTERA-2 cells after treatment with 6-gingerol; results suggested a significant decrease in the Fe2+ ion concentration following 6-gingerol treatment (**Figure 5B**). These results indicated an upregulation in iron transport by the conversion of Fe2+ to Fe3+, which highlighted the conversion of total iron for iron metabolism.

To confirm this, we analyzed the expression levels of proteins responsible for iron transport, and the results showed downregulated expression of TFR1 and ferroportin (FPN1), which transports iron (**Figure 5C**). Also, we found that 6-gingerol suppressed the expression of DMT1 and STEAP3, helping iron conversion. These results suggested a role of iron metabolism in the anti-cancer activity of 6-gingerol against embryonic CSCs. Next, we analyzed the role of iron metabolism in PTEN induction and p53/PD-L1 signaling. For this, we added iron sulfate (FeSO_4_) to NCCIT and NTERA-2 cells with or without 6-gingerol treatment. Supplementation with FeSO_4_ resulted in suppressed PTEN and p53 expression, which was accompanied by an elevation in PD-L1 expression (**Figure 5D**). The addition of 6-gingerol reversed these expression patterns, which suggested a role for iron metabolism in PTEN-mediated p53 and PD-L1 expressions following 6-gingerol treatment in embryonic CSCs.

### 6-Gingerol Downregulates miR-20b, miR-21, and miR-130b

**Figure 6:**
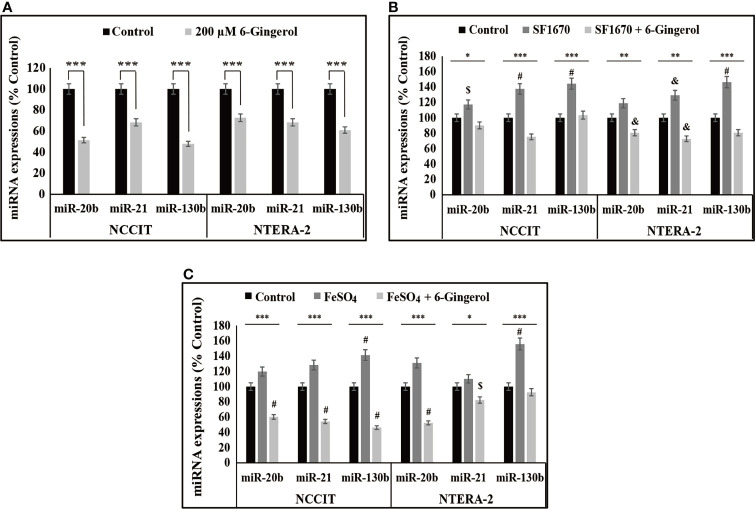
6-Gingerol regulated expression of miR-20b, miR-21, and miR-130b. **(A)** Repre-sentative real-time qPCR analysis of miR-20b, miR-21, and miR-130b transcripts following treatment with 200 μM 6-gingerol for 48 h in embryonic CSCs. Then, Cp values were normalized to U6 mRNA. Controls were set at 100. ***p < 0.001. (Student’s t-test). **(B)** Representative real-time qPCR of miR-20b, miR-21, and miR-130b transcripts following treatment with 2 μM SF1670 with or without 200 μM 6-gingerol for 48 h in NCCIT and NTERA-2 cells. Cp values were normalized to *U6* mRNA. Controls were set at 100. *p < 0.05, **p < 0.01, and ***p < 0.001. (ANOVA test), ^$^ *p* < 0.05 vs. control, ^&^ *p* < 0.01 *vs*. control, and ^#^ *p* < 0.001 *vs*. control. **(C)** Representative real-time qPCR of miR-20b, miR-21, and miR-130b in NCCIT and NTERA-2 cells treated with 200 μM 6-gingerol for 48 h, followed by 200 μM FeSO_4_ for an additional 48 h. Cp values were normalized to *U6* mRNA. Controls were set at 100. *p < 0.05 and ***p < 0.001 (ANOVA test). ^$^ *p* < 0.05 *vs*. control. ^#^ *p* < 0.001 *vs*. control.

We found that iron metabolism and PTEN/p53/PD-L1 signaling may play a role in the induction of apoptosis by 6-gingerol in embryonic CSCs. Here, we analyzed the potential role of microRNA in the apoptotic activity of 6-gingerol and the role of iron metabolism in microRNA expression in the presence of 6-gingerol. First, we measured the expression levels of miR-20b, miR-21, and miR-130b, which play key roles in PTEN-mediated PD-L1 expression. The mRNA analysis showed a downregulation of miR-20b, miR-21, and miR-130b following 6-gingerol treatment in NCCIT and NTERA-2 cells (**Figure 6A**). These data indicated a potential role for these microRNAs in apoptosis induction by 6-gingerol. To confirm this activity, we used the PTEN inhibitor SF1670 and analyzed the expression of these microRNAs in the presence of 6-gingerol (**Figure 6B**). The results showed elevated expression of miR-20b, miR-21, and miR-130b with PTEN inhibitor treatment, which was downregulated by 6-gingerol treatment. These results suggested a role for PTEN in regulating these microRNAs.

Next, we verified the role of iron metabolism in the expression of miR-20b, miR-21, and miR-130b by supplementing FeSO_4_ with or without 6-gingerol. These results also showed an increase in the expression of miR-20b, miR-21, and miR-130b following FeSO_4_ treatment, which were reversed by the addition of 6-gingerol in both NCCIT and NTERA-2 cells (**Figure 6C**). These results contributed to evidence that iron metabolism plays a role in the induction of cell death by regulating microRNA expression following 6-gingerol treatment.

## Discussion

Anti-cancer activity partially depends on how a drug impacts cancer cells and prevent cancer recurrence by targeting CSCs. Cancer recurrence is one of the major challenges for many chemotherapeutic drugs, as they successfully suppress tumor progression but may not have targeted CSCs (37). The use of natural compounds as cancer therapeutics is promising for long-term use as they may reduce side effects and target both cancer cells and CSCs. 6-Gingerol successfully suppressed the expression of CSC markers and Wnt/β-catenin signaling in NCCIT and NTERA-2 cells, suggesting that 6-gingerol might target cancer cells as well as CSCs.

A natural compound that can induce DDR, thereby inducing cell cycle arrest and apoptosis in cancer cells, should be considered as a candidate for further studies. Previous studies showed that 6-gingerol can induce cell cycle arrest and apoptosis against several cancer types (28, 34, 38). The anti-cancer activity of a natural compound also depends on its capability to induce ROS generation (39). We found that 6-gingerol elevated the expression of iNOS at the transcriptional and translational levels so that iNOS induction leads to ROS generation (28). As a result, treatment with 6-gingerol significantly elevated cellular and mitochondrial ROS, which may hint for anti-cancer activity of 6-gingerol. We demonstrated 6-gingerol induction of DDR by introducing DNA double-strand breaks. ATM or ATR kinases sense DNA damage and are central regulators in the response to DNA damage (40). Next, our results also showed an elevation in the expression levels of these kinases, which then activated p53 expression to proceed to cell cycle arrest and apoptosis. This leads to the phosphorylation of other substrates such as BRCA1, CHK1, or CHK2 (41, 42). Prolonged DNA damage results in cell cycle arrest, and p53 is a key factor in this mechanism (43). We showed that 6-gingerol successfully induced DDR, G0/G1 cell cycle arrest, and p53 expression in NCCIT and NTERA-2 cells. These results suggested a possible apoptosis induction in embryonic CSCs following 6-gingerol treatment.

The apoptosis pathway is divided into intrinsic and extrinsic pathways. Mitochondria play a central role in the intrinsic pathway through the p53-dependent upregulation of BAX and downregulation of BCL-2, which promotes the release of cytochrome c from mitochondria to the cytosol. The presence of cytochrome c in the cytosol activates caspase proteins to induce apoptosis (44, 45). Flow cytometry results suggested 6-gingerol induction of apoptosis in NCCIT and NTERA-2 cells. An analysis of the apoptosis induction pathway following 6-gingerol treatment matched the expectations of intrinsic pathway activation; we observed an upregulation of BAX, cleaved caspase 9, and cytochrome c expression as well as downregulated expression of BCL-2 and BCL-xL. These results provide evidence that 6-gingerol induces mitochondrial apoptosis.

Iron metabolism can induce ROS generation that might lead to the DDR, thereby causing cell cycle arrest and apoptosis. Iron metabolism also plays a crucial role in tumor progression; control of iron homeostasis could be a key target in anti-cancer activity (46). Adding 6-gingerol to NCCIT and NTERA-2 cells showed a decrease in the amount of cellular Fe2+ and a release of total iron into spent medium, suggesting the inhibition of iron transport in NCCIT and NTERA-2 cells by 6-gingerol. Molecular analysis of the proteins responsible for iron homeostasis provided additional proof for our hypothesis of iron metabolism inhibition by 6-gingerol. Hence, ROS generation and an inhibition of iron metabolism might contribute to apoptosis induction by 6-gingerol. The role of iron metabolism was also confirmed by the activity of FeSO_4_ on the expression of the tumor suppressor protein PTEN. PTEN exhibits an essential role in embryonic development, and it may regulate the renewal and differentiation of stem cells by mediating GSK3-β expression, a key factor in Wnt/β-catenin signaling (47). A loss of PTEN upregulates PD-L1 expression through the activation of the PI3K/AKT pathway (48, 49). The suppression of p53 also involves the regulation of PD-L1 expression through PI3K/AKT signaling (50). Our results demonstrated an increase in the expression patterns of PTEN and p53, with a decrease in PI3K/AKT signaling and PD-L1 expression following 6-gingerol treatment of NCCIT and NTERA-2 embryonic CSCs. We also confirmed the role of PTEN in PD-L1 expression in both cell types using a specific PTEN inhibitor and demonstrated the ability of 6-gingerol to regulate these signaling pathway by reversing the effect of the PTEN inhibitor.

The suppression of PD-L1 depends on the regulation of microRNAs (miRNAs). miRNAs are a family of small noncoding RNAs that play vital roles in cancer by regulating other signaling pathways, inhibiting mRNA translation, and promoting mRNA degradation, all of which result in the post-transcriptional modification of gene expression (51). Many microRNAs such as miR-34a, miR-197, and miR-200 are involved in the expression of PD-L1 (51). Among others, miR-20, miR-21, and miR-130 mediate PD-L1 expression by regulating PTEN expression (52). Treatment of NCCIT and NTERA-2 cells with 6-gingerol showed a downregulation of miR-20b, miR-21, and miR-130b expression. Next, the regulation of these miRNAs was confirmed using a PTEN inhibitor and 6-gingerol treatment. To analyze the role of iron metabolism in these miRNAs’ expression, we used FeSO_4_ to treat NCCIT and NTERA-2 cells with and without 6-gingerol. The expression of miR-20b, miR-21, and miR-130b were increased with FeSO_4_ supplementation, which was successfully reversed by 6-gingerol treatment. It is evident that the inhibition of iron metabolism contributes to the ability of these microRNAs to elevate PTEN expression, which thereby inhibits PD-L1 expression to promote the intrinsic apoptosis pathway.

This study demonstrated that a natural sulfur-containing compound, 6-gingerol, targets embryonic CSCs by inhibiting CSC markers and Wnt/β-catenin signaling in NCCIT and NTERA-2 cells. Furthermore, 6-gingerol induced the intrinsic pathway of apoptosis in these embryonic CSCs through the induction of PTEN, thereby inhibiting PD-L1 expression. Iron metabolism plays a vital role in the elevation of PTEN expression through ROS generation, thereby regulating PI3K/AKT/p53 signaling to inhibit PD-L1 expression and suppress the expression of miR-20b, miR-21, and miR-130b. Altogether, 6-gingerol is a candidate for adjuvant chemotherapy, as it may suppress cancer recurrence by targeting CSCs.

## Data Availability Statement

The original contributions presented in the study are included in the article/**Supplementary Material**. Further inquiries can be directed to the corresponding authors.

## Author Contributions

K-JJ and SWB designed the experiments. NS, DYK, and ESJ performed all the experiments. J-ML served as scientific advisors and participated in technical editing of the manuscript. NS and K-JJ wrote the manuscript. NS, DYK, SWB, and K-JJ analyzed the data. All authors helped in revising the manuscript and approved the final version for publication. All authors read and agreed to the published version of the manuscript.

## Funding

This work was supported by the research grant of Jeju National University in 2021.

## Conflict of Interest

Author J-ML was employed by company SK Bioscience.

The remaining authors declare that the research was conducted in the absence of any commercial or financial relationships that could be construed as a potential conflict of interest.

## Publisher’s Note

All claims expressed in this article are solely those of the authors and do not necessarily represent those of their affiliated organizations, or those of the publisher, the editors and the reviewers. Any product that may be evaluated in this article, or claim that may be made by its manufacturer, is not guaranteed or endorsed by the publisher.
