# Supplementary material for: Pivotal Role of Iron Homeostasis in the Induction of Mitochondrial Apoptosis by 6-Gingerol Through PTEN Regulated PD-L1 Expression in Embryonic Cancer Cells
Source: Front Oncol. 2021 Nov 3;11:781720. doi: 10.3389/fonc.2021.781720 (PMC8595921; doi:10.3389/fonc.2021.781720)
Supplement: Supplementary file 1 [file DataSheet_1.docx]

Supplementary Material

## Supplementary Figures


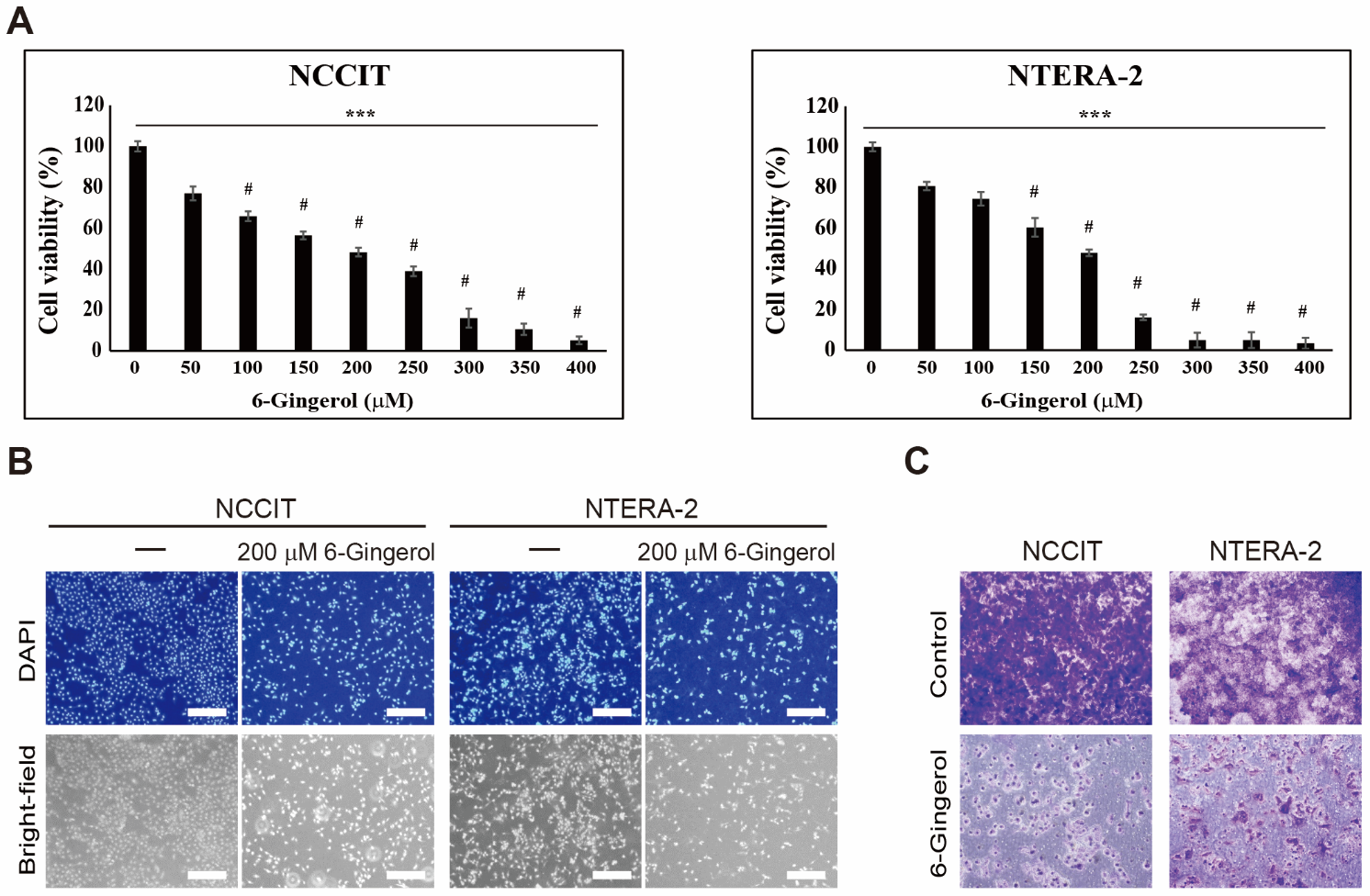


**Supplementary Figure 1.** 6-Gingerol inhibited embryonic CSC proliferation. (A) MTT assay showing cell viability inhibition of NCCIT and NTERA-2 cells with increasing concentrations of 6-gingerol for 48 h. Data are representative of three independent experiments. **** p* < 0.001 (*ANOVA* test). *# p* < 0.001 *vs*. control. (B) 6-Gingerol induced nuclear aberrations in embryonic CSCs. Phase-contrast microscopy images of abnormal nucleus formation induced in NCCIT and NTERA-2 cells by 48 h treatment with 200 µM 6-gingerol. Representative photographs are presented (scale bar: 200 μm). (C) Matrigel invasion assay illustrated that 200 µM 6-gingerol for 48 h could inhibit the invasion of NCCIT and NTERA-2 cells. Scale bar, 100 μm.

**
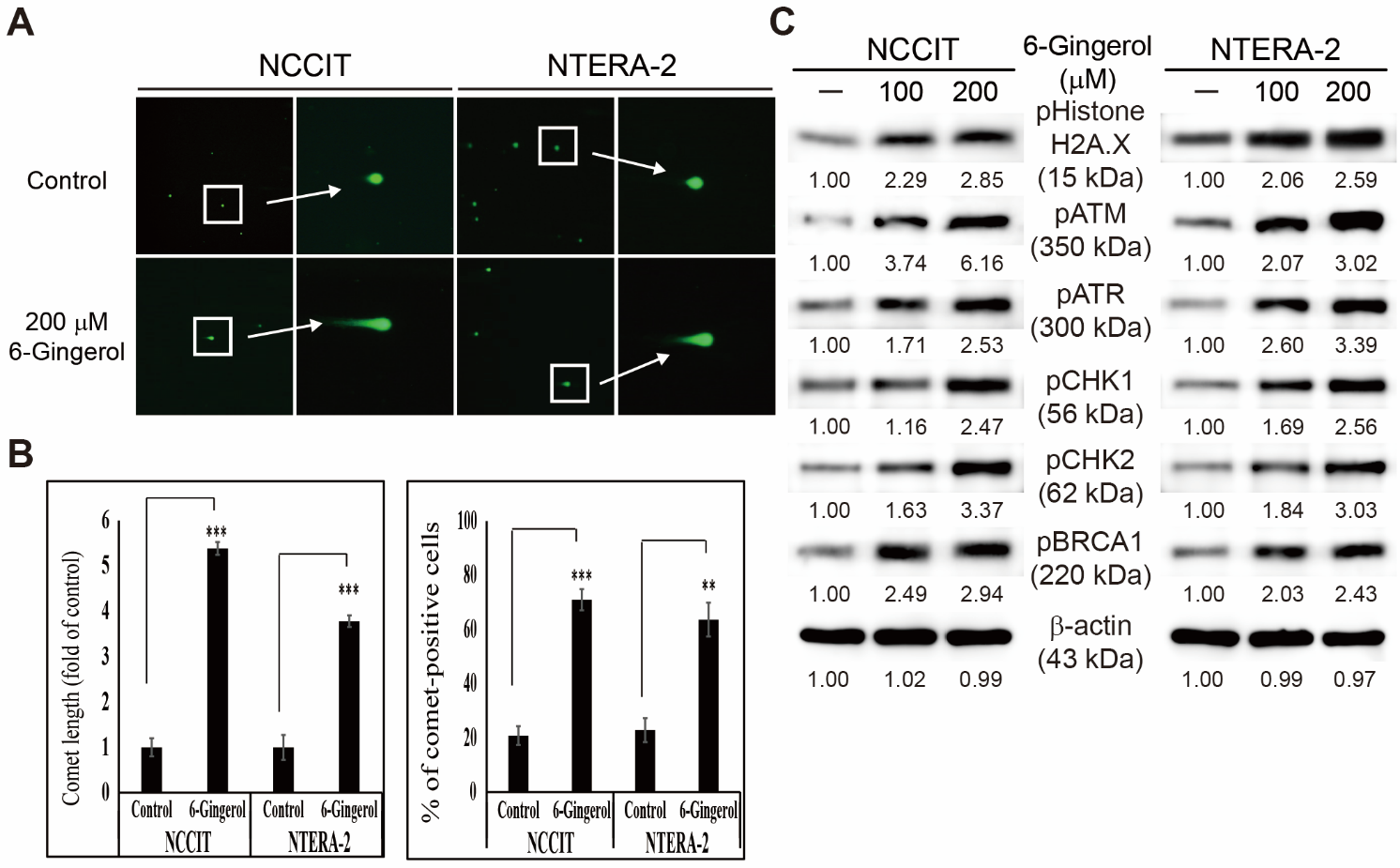
**

**Supplementary Figure 2.** 6-Gingerol induces DDR in embryonic CSC. (A) Comet assay images from ﬂuorescent microscopy at 10× and 40× magnification show fragmented DNA migration from the nucleoid body, which forms a comet tail. Images of NCCIT and NTERA-2 cells after 48 h treatment with 200-μM 6-gingerol. (B) A plot of comet length was analyzed as the fold change versus the control, and the percentage of comet-positive cells following 6-gingerol treatment in embryonic CSCs. **** p* < 0.001 (*Student’s t*-test). (C) Western blot of phospho-histone H2A.X, ATM, ATR, CHK1, CHK2, and BRCA1 in NCCIT and NTERA-2 cells treated for 48 h with 100 or 200 μM of 6-gingerol. Expression levels were estimated by densitometry and normalized to β-actin. Data were obtained in triplicate.

**
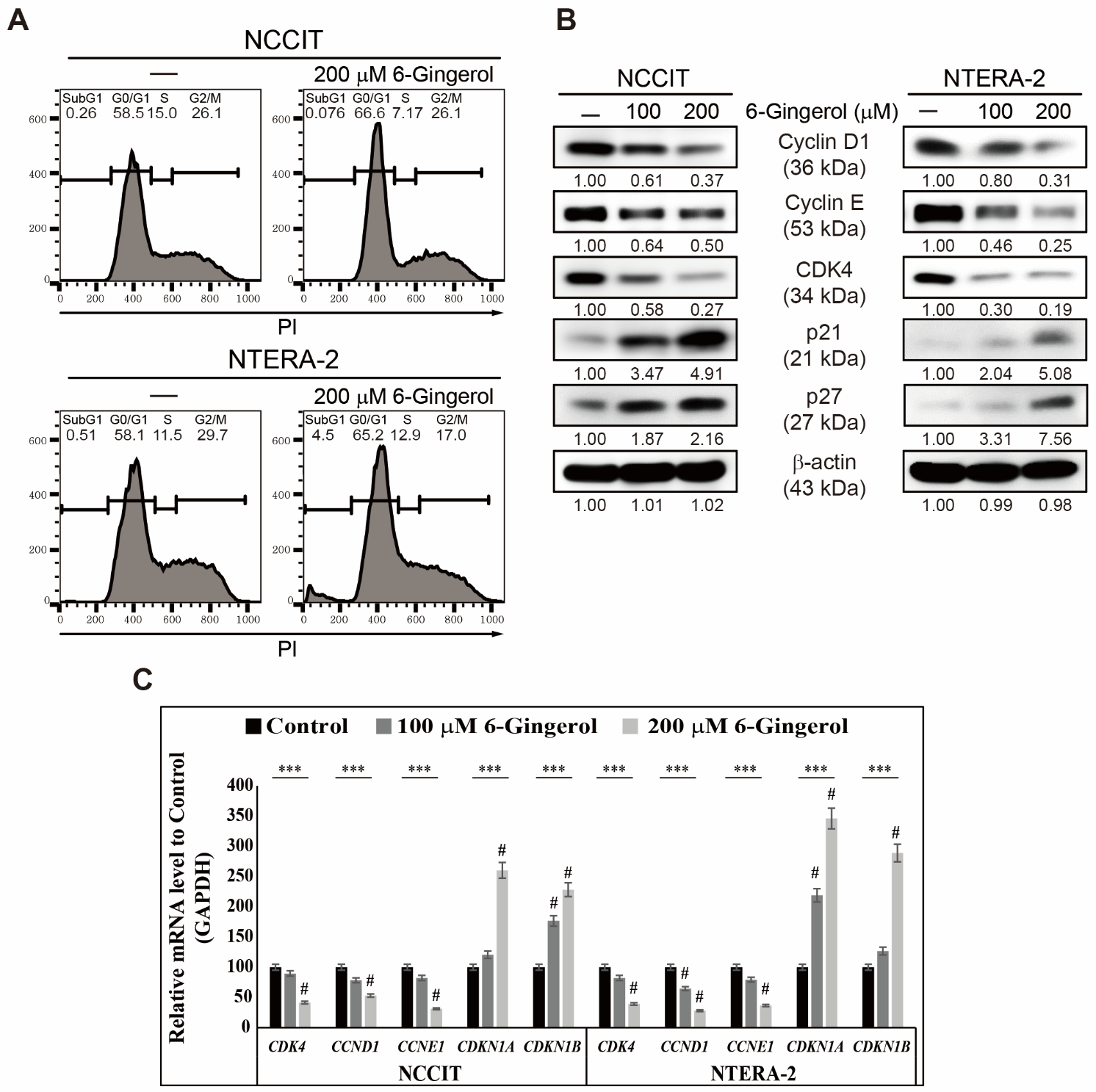
**

**Supplementary Figure 3.** 6-Gingerol induces G0/G1 cell cycle arrest. (A) Flow cytometry of PI staining shows the cell cycle distribution in NCCIT and NTERA-2 cells after 48 h treatment with 200 μM 6-gingerol. (B) Western blot of cyclin D1, cyclin E, CDK4, p21, and p27 in NCCIT and NTERA-2 cells after a 48-h incubation with 100 or 200 μM 6-gingerol. Expression levels were estimated by densitometry and normalized to β-actin. Data were obtained in triplicate. (C) Representative RT-qPCR of the cell cycle checkpoint genes *CCND1, CCNE1, CDK4, CDKN1A,* and *CDKN1B*; Cp values were normalized to *GAPDH* mRNA. Controls are set to 100. **** p* < 0.001 (*ANOVA* test). # *p* < 0.001 *vs*. control.
